# Red cell distribution width-to-albumin ratio as a potential biomarker for short-term mortality risk in critically ill patients with cerebral hemorrhage: a retrospective study with dual-cohort validation

**DOI:** 10.3389/fnut.2026.1804846

**Published:** 2026-03-27

**Authors:** Zirong Gao, Yijie Gao, Li Huang, Meilan Fan, Cheng Zha, Shanshan Yu

**Affiliations:** 1 Department of Neuro Intensive Care Unit, The Affiliated Jinyang Hospital of Guizhou Medical University, Guiyang, China; 2 Department of Neuro Intensive Care Unit, The Second People's Hospital of Guiyang (Jinyang Hospital), Guiyang, China; 3 Medical College of Jinzhou Medical University, Jinzhou, China; 4 Zhejiang Provincial People's Hospital Bijie Hospital, Bijie, China

**Keywords:** intracerebral hemorrhage, machine learning algorithms, RAR, risk prediction model, short-term mortality

## Abstract

**Background:**

The red cell distribution width-to-albumin ratio (RAR) is a composite biomarker integrating inflammatory, nutritional, and stress status; however, its association with short-term prognosis in patients with intracerebral hemorrhage (ICH) remains unclear.

**Methods:**

This study was a retrospective dual-cohort study. A total of 2,327 ICH patients from the MIMIC-IV database were included as the derivation cohort, and 428 patients from a tertiary hospital were collected as the external validation cohort. The association between RAR and outcomes was analyzed using multivariable Cox regression, with restricted cubic splines employed to examine non-linear relationships. Multiple machine learning algorithms were utilized to screen key prognostic variables, and a logistic regression-based risk prediction model was constructed. Its discriminative ability and stability were validated in both internal and external cohorts.

**Results:**

After adjusting for multiple confounders, including demographic characteristics, comorbidities, disease severity, and treatment measures, a higher RAR level remained an independent risk factor for 28-day ICU mortality (adjusted HR = 1.17, 95% CI: 1.08–1.27) and 28-day in-hospital all-cause mortality (adjusted HR = 1.14, 95% CI: 1.05–1.23) in ICH patients. Restricted cubic spline analysis further indicated a significant non-linear dose-response relationship between RAR and these outcomes (*P* for non-linearity < 0.05). In addition, incorporating RAR significantly improved the predictive performance of six traditional critical illness scoring systems, including APACHE II, SOFA, and SAPS II (AUC improvement ranging from 0.016 to 0.188; all DeLong tests *P* < 0.01). Using five machine learning algorithms, we identified seven key variables—age, RAR, INR, total bilirubin, blood urea nitrogen, aspartate aminotransferase, and systolic blood pressure—to construct a short-term mortality risk prediction model for ICH. This model demonstrated robust discriminative ability in the internal training, internal validation, and external validation sets (AUC values of 0.761, 0.723, and 0.723, respectively), outperforming conventional scoring systems.

**Conclusion:**

RAR is an independent predictor of short-term mortality risk in patients with ICH. The prediction model incorporating RAR exhibits good discriminative ability and cross-cohort stability, offering a practical tool for early identification of high-risk patients and optimization of management strategies. However, this study has certain limitations, including its retrospective design, limited sample size and single-center source for external validation, and lack of neuroimaging data (e.g., hematoma location/volume). Future prospective multi-center studies are needed to further validate its clinical value.

## Background

Intracerebral hemorrhage (ICH) refers to the pathological process in which blood extravasates into the brain parenchyma due to rupture of cerebral blood vessels (1, 2). Globally, the annual incidence of non-traumatic ICH is approximately 3.5 million cases, with a high mortality rate: up to 40% within the first month after onset and rising to 54% within one year (3). Moreover, most survivors often experience long-term functional and cognitive impairment, imposing a substantial burden on society and families (3–5). Therefore, timely clinical intervention and early prognosis assessment are crucial for reducing mortality, mitigating disease severity, and minimizing complications. Against this background, there is an urgent need to develop biomarkers that can accurately predict poor outcomes in ICH. However, although some existing scoring systems have been shown to correlate with ICH patient outcomes, they are often cumbersome and inconvenient to use, making them less than ideal as practical prediction tools in clinical practice (6–8). Consequently, there is a pressing demand for biomarkers with good predictive performance and ease of application to assist clinicians in early identification of high-risk patients and to guide treatment decisions.

Serum albumin, the most abundant circulating protein in blood, serves as an important biomarker for assessing nutritional status and systemic inflammatory response. It possesses multiple physiological functions, including anti-inflammatory, antioxidant, and maintenance of colloidal osmotic pressure (9, 10). Red cell distribution width (RDW), a routine hematologic parameter, reflects heterogeneity in red blood cell volume. Elevated RDW is often associated with pathological processes such as inflammation, oxidative stress, and malnutrition (11, 12). Studies have shown that both low albumin levels and high RDW values are independently associated with poor outcomes in ICH patients (13–16). However, a single indicator is susceptible to the influence of the patient's baseline physiological state and clinical interventions, limiting its predictive stability.

Given the pathophysiological complexity following ICH, single biomarkers often fail to fully capture the interplay between systemic inflammatory stress and nutritional depletion (1–4). An elevated RDW not only reflects inflammation-induced dysregulation of erythropoiesis but may also indicate reduced deformability of erythrocytes, potentially exacerbating cerebral microcirculatory disturbances and secondary ischemic injury. Conversely, hypoalbuminemia signifies not merely malnutrition but also an impaired capacity for antioxidant defense, maintenance of blood-brain barrier integrity, and regulation of cerebral edema (9, 10, 17, 18). The RDW-to-albumin ratio (RAR) integrates these two dimensions and may serve as a composite marker reflecting the balance between pro-inflammatory injury and endogenous repair capacity (19–21). Consequently, a high RAR state could synergistically exacerbate secondary brain injury by promoting neuroinflammation, microvascular dysfunction, and cerebral edema, ultimately leading to an increased risk of short-term mortality.

However, while existing studies have established an association between RAR and long-term prognosis (90 or 365 days) in ICH patients (22), such extended timeframes introduce numerous confounding factors, such as post-discharge care quality, complications, and underlying diseases, which undermine the direct relevance of RAR as an acute-phase marker of inflammation/nutritional status for immediate clinical decision-making. Therefore, this study aimed to focus the observation endpoint on ICU and in-hospital outcomes within 28 days. This period represents a critical window of intensive clinical intervention that largely determines short-term survival and functional status. This approach is intended to provide a novel and practical tool for early risk stratification.

## Methods

### Data sources

This study utilized two retrospective cohorts. The derivation cohort was derived from the publicly accessible Medical Information Mart for Intensive Care IV (MIMIC-IV) database, version 2.2 (23). This database contains information on 196,527 adults admitted to the Beth Israel Deaconess Medical Center between 2008 and 2019. Access to the database was granted under an approved protocol. The Institutional Review Board of the Massachusetts Institute of Technology approved the use of the MIMIC-IV database for research and waived the requirement for informed consent due to the retrospective nature of the study and the use of de-identified data. The external validation cohort consisted of ICH patients treated in the Neuro-Intensive Care Unit of Zhejiang Provincial People's Hospital Bijie Hospital. After approval by the hospital ethics committee, this cohort was enrolled between January 2015 and January 2025; the committee waived the requirement for individual informed consent owing to the retrospective study design.

### Study population

When extracting data from the MIMIC-IV database, we used PostgreSQL (v13.7.2) and Navicat Premium (v16) to perform structured queries. The study subjects were all adult patients (age ≥ 18 years) diagnosed with non-traumatic intracerebral hemorrhage (ICH) based on ICD-9 code 431 and ICD-10 codes I610–I619 and I62.9. To ensure data completeness and analytical reliability, the following exclusion criteria were applied: (1) patients who died within 24 h of ICU admission, as their clinical records might be incomplete or survival time could be negative; (2) patients with multiple ICU admissions—only the first admission for ICH was included to avoid data duplication; (3) patients without key laboratory indicators (including RDW and albumin) measured on the first day of ICU admission, as these data are essential for the analysis in this study.

### Variable extraction and processing

Data extraction was performed on the PostgreSQL (v13.7.2) and Navicat Premium (v16.0) platforms using Structured Query Language (SQL). The extracted variables were grouped into six categories: (1) demographic characteristics; (2) comorbidities; (3) vital signs; (4) laboratory parameters; (5) disease severity scores; and (6) treatment measures. A detailed list of variables is provided in Supplementary Tables 1, S1, S2. For missing data, only variables with a missing rate below 30% were imputed. We used the “mice” package (v3.16.0) in R, based on a random-forest model, to perform multiple imputation. The imputation model included all variables involved in the primary analysis: the continuous variables to be imputed, fully observed exposure variables (RAR and its components), the complete outcome variable (28-day mortality), and other complete categorical and continuous covariates. In this study, the primary endpoint was 28-day ICU mortality, and the secondary endpoint was 28-day in-hospital all-cause mortality.

### Association between RAR and endpoints

Following hypothesis testing, Cox proportional hazards regression models were used to analyze the association between RAR and clinical endpoints. To control for potential confounders, three progressively adjusted models were constructed: Model 1 was unadjusted; Model 2 was adjusted for demographic characteristics (age, sex, race, body mass index) and major comorbidities; Model 3 was further adjusted for clinical variables that differed significantly between survivors and non-survivors, including disease severity scores, key laboratory parameters, and treatment measures (see Supplementary Tables S1–S3 for details). To reduce the impact of multicollinearity on model stability, variance inflation factors (VIFs) were calculated for all variables in Model 3, and variables with VIF > 5 were removed. Subsequently, restricted cubic splines (RCS) were used to fit the relationship between RAR and endpoints, with knots set at the 5th, 35th, 65th, and 95th percentiles, to explore potential non-linear trends.

### Incremental effect of RAR

RAR was incorporated into six traditional critical illness scoring systems to build multivariable Cox proportional hazards models. Based on the regression coefficients of each variable, a composite risk score was calculated using the formula (β1 × variable1) + (β_2_ × variable_2_). The predictive performance of each model for adverse outcomes was evaluated by plotting receiver operating characteristic (ROC) curves and calculating the area under the curve (AUC). DeLong's test was employed to compare the predictive ability of the models before and after adding RAR, to determine whether the improvement was statistically significant.

### Subgroup and interaction analyses

To verify the stability of the relationship between RAR and adverse outcomes in ICH, prespecified subgroup analyses and interaction tests were performed. Subgroups were defined according to key characteristics such as age, sex, race, and baseline comorbidities. By testing the statistical significance of interaction terms (P for interaction), we assessed whether the effect of RAR was consistent across subgroups. If no significant interactions were observed (typically *P* > 0.05), this would support the stability of the relationship across different populations; if significant interactions existed, this would suggest heterogeneity. All analyses were based on a prespecified protocol to control for bias due to multiple comparisons.

### Screening of important prognostic features

In the internal cohort, we first randomly divided the data into training and validation sets at a 7:3 ratio. During the training phase, the following five machine learning algorithms were jointly applied to screen for variables closely related to ICU mortality: Boruta algorithm (confidence level *p* < 0.01, maximum iterations = 100, with Bonferroni correction), random forest (100 trees, maximum depth = 3), gradient boosting machine based on Cox loss function (learning rate = 0.1, boosting rounds = 100), Lasso regression (LogLambda min = −4.841), and support vector machine (regularization parameter = 1.0, radial basis function kernel). Finally, variables identified as important by all five algorithms were designated as core prognostic features for this cohort.

### Definition of key variables

Consensus features were defined as variables identified as important by all five machine learning algorithms. The specific identification process was as follows: features “confirmed” by the Boruta algorithm were selected; the top 10 important features were chosen by random forest and GBM, respectively; Lasso regression retained features with non-zero coefficients after 10-fold cross-validation; and SVM-RFE, combined with 5-fold cross-validation, selected the optimal feature subset. Subsequently, a Venn diagram was used to identify the intersection of features selected by all five methods. This consensus strategy, through cross-validation across multiple algorithms, effectively reduces the selection bias that may arise from a single method and enhances the reliability and clinical interpretability of the core features.

### Risk prediction modeling and validation

In this study, the screened key variables were included in the final multivariable logistic regression (Cox) risk model. The regression coefficients (β) reflected the change in log hazard ratio per unit increase in each variable after adjusting for other factors. Based on this, each patient's risk score was calculated using the linear combination formula: Risk score = Σ(β? × variable?). The discriminative ability of the model was evaluated by ROC curves and AUC, and its predictive performance and stability were tested in an independent external validation cohort.

### Statistical analysis

For data visualization and comparative analysis, patients were divided into low-, medium-, and high-RAR groups based on tertiles. This grouping was used solely for descriptive and exploratory purposes and did not presuppose any clinical risk threshold. Continuous variables are presented as mean ± standard deviation; after testing for normality and homogeneity of variance, Student's *t*-test or analysis of variance (ANOVA) was used for between-group comparisons. Categorical variables are described as frequency (percentage), and between-group comparisons were performed using Pearson's chi-square test or Fisher's exact test as appropriate. All analyses were performed with R software (version 4.5.1), and a two-sided *p* < 0.05 was considered statistically significant.

## Result

### Baseline patient data

A total of 2,327 critically ill patients meeting the inclusion criteria were analyzed. Baseline characteristics stratified by the Red cell Distribution Width to Albumin Ratio (RAR) are presented in Table 1. Compared to the low-RAR group, patients in the high-RAR group had a greater burden of comorbidities, required more intensive therapeutic interventions, and exhibited less stable vital signs, including faster heart rates and lower systolic, diastolic, and mean arterial pressures. Several laboratory parameters also differed significantly between the groups. The high-RAR group was associated with elevated levels of RDW, WBC, INR, PT, PTT, ALT, AST, total bilirubin, creatinine, and blood urea nitrogen, but lower levels of albumin, serum calcium, hemoglobin, red blood cell count, and platelet count. Clinical severity scores (SOFA, APS III, SAPS II, OASIS, APACHE II) increased significantly with higher RAR levels. Regarding clinical outcomes, the high-RAR group had significantly higher in-hospital mortality (26.7% vs. 7.10%, *p* < 0.001) and ICU mortality (28.3% vs. 7.10%, *p* < 0.001) than the low-RAR group, along with longer hospital and ICU stays.

**Table 1:** Baseline data of patients in RAR grouping (internal queue).

	ALL	Low	Moderate	High	*p*-value
	***N*** = **2,327**	***N*** = **775**	***N*** = **777**	***N*** = **775**	
RAR	4.34 (1.14)	3.34 (0.27)	4.11 (0.23)	5.56 (1.10)	0.000
Age	65.2 (17.6)	61.9 (17.8)	66.7 (17.2)	66.9 (17.2)	< 0.001
Gender:	1,371 (58.9%)	469 (60.5%)	454 (58.4%)	448 (57.8%)	0.525
Race:	1,370 (58.9%)	477 (61.5%)	472 (60.7%)	421 (54.3%)	0.007
Weight	78.3 (21.1)	78.0 (19.5)	78.6 (21.3)	78.4 (22.4)	0.865
HTN:	1,245 (53.5%)	459 (59.2%)	449 (57.8%)	337 (43.5%)	< 0.001
AKI:	509 (21.9%)	73 (9.42%)	152 (19.6%)	284 (36.6%)	< 0.001
CKD:	254 (10.9%)	33 (4.26%)	88 (11.3%)	133 (17.2%)	< 0.001
DM:	519 (22.3%)	129 (16.6%)	180 (23.2%)	210 (27.1%)	< 0.001
HLD:	699 (30.0%)	226 (29.2%)	247 (31.8%)	226 (29.2%)	0.427
IHD:	451 (19.4%)	103 (13.3%)	150 (19.3%)	198 (25.5%)	< 0.001
COPD:	199 (8.55%)	34 (4.39%)	73 (9.40%)	92 (11.9%)	< 0.001
SOFA	3.98 (2.93)	2.90 (2.00)	3.74 (2.48)	5.30 (3.55)	< 0.001
APSIII	41.0 (18.7)	34.6 (14.3)	39.8 (16.7)	48.6 (21.6)	< 0.001
SAPSII	34.6 (12.3)	29.4 (10.4)	34.7 (11.0)	39.6 (13.2)	< 0.001
OASIS	32.2 (7.80)	29.5 (7.31)	32.6 (7.18)	34.4 (8.10)	< 0.001
GCS	12.6 (3.08)	12.6 (2.85)	12.5 (3.08)	12.7 (3.30)	0.484
APACHEII	15.5 (6.65)	12.6 (5.26)	15.2 (5.92)	18.7 (7.19)	< 0.001
HR	84.7 (18.9)	81.5 (16.9)	83.9 (18.3)	88.9 (20.8)	< 0.001
NBPS	132 (24.1)	135 (21.8)	134 (24.0)	127 (25.6)	< 0.001
NBPD	74.0 (18.4)	75.9 (16.9)	74.9 (19.5)	71.2 (18.2)	< 0.001
NBPM	88.6 (18.1)	90.7 (16.6)	90.0 (18.8)	85.2 (18.4)	< 0.001
RR	18.8 (5.52)	18.0 (4.70)	18.6 (5.30)	19.6 (6.33)	< 0.001
Spo2	102 (202)	97.8 (2.71)	97.5 (3.04)	110 (349)	0.107
HCT	35.0 (6.14)	37.7 (4.70)	35.6 (5.57)	31.8 (6.49)	< 0.001
Hb	11.7 (2.13)	12.7 (1.60)	11.9 (1.88)	10.4 (2.16)	< 0.001
PLT	209 (95.3)	219 (77.3)	212 (88.0)	195 (115)	< 0.001
RDW	14.3 (1.81)	13.1 (0.76)	14.0 (1.09)	15.6 (2.18)	< 0.001
RBC	3.86 (0.75)	4.14 (0.58)	3.92 (0.69)	3.51 (0.82)	< 0.001
WBC	12.1 (12.6)	11.3 (4.63)	11.5 (5.25)	13.5 (20.6)	0.015
ALB	3.41 (0.57)	3.95 (0.31)	3.41 (0.28)	2.87 (0.46)	0.000
AG	14.7 (3.87)	14.9 (3.45)	14.5 (3.48)	14.8 (4.56)	0.095
Ca	8.53 (0.77)	8.74 (0.66)	8.53 (0.72)	8.32 (0.86)	< 0.001
Cl	104 (5.87)	103 (4.71)	104 (5.79)	104 (6.84)	< 0.001
Glu	149 (70.5)	143 (59.1)	149 (61.6)	154 (87.1)	0.006
K	4.02 (0.68)	3.99 (0.62)	3.98 (0.66)	4.08 (0.76)	0.009
Na	139 (4.93)	139 (3.94)	139 (4.81)	139 (5.86)	0.506
PH	7.40 (0.08)	7.42 (0.07)	7.40 (0.08)	7.38 (0.10)	< 0.001
INR	1.26 (0.43)	1.17 (0.27)	1.23 (0.49)	1.36 (0.46)	< 0.001
PT	13.9 (5.17)	12.9 (2.72)	13.6 (5.18)	15.0 (6.62)	< 0.001
PTT	30.8 (14.5)	29.1 (10.5)	30.1 (14.0)	33.2 (17.8)	< 0.001
ALT	63.6 (307)	44.6 (84.6)	65.2 (308)	81.0 (425)	0.016
AST	92.2 (513)	55.3 (138)	91.9 (482)	130 (731)	0.004
TB	1.00 (2.24)	0.70 (0.58)	0.78 (1.13)	1.51 (3.61)	< 0.001
CRE	1.15 (1.31)	0.91 (0.65)	1.09 (1.08)	1.46 (1.85)	< 0.001
URE	20.3 (17.2)	15.2 (7.40)	19.1 (14.9)	26.5 (23.4)	< 0.001
Mg	1.90 (0.35)	1.90 (0.28)	1.88 (0.34)	1.92 (0.43)	0.078
ventilation:	1,854 (79.7%)	553 (71.4%)	633 (81.5%)	668 (86.2%)	< 0.001
CRRT:	75 (3.22%)	2 (0.26%)	11 (1.42%)	62 (8.00%)	< 0.001
SA:	1,511 (64.9%)	419 (54.1%)	522 (67.2%)	570 (73.5%)	< 0.001
VP:	857 (36.8%)	171 (22.1%)	273 (35.1%)	413 (53.3%)	< 0.001
GC:	554 (23.8%)	172 (22.2%)	174 (22.4%)	208 (26.8%)	0.052
Hosp dead	353 (15.2%)	55 (7.10%)	91 (11.7%)	207 (26.7%)	< 0.001
Hosp time	18.0 (19.3)	14.1 (13.2)	17.9 (18.7)	22.1 (23.8)	< 0.001
ICU dead	368 (15.8%)	55 (7.10%)	94 (12.1%)	219 (28.3%)	< 0.001
ICU time	8.31 (9.55)	6.54 (6.77)	8.38 (8.46)	10.00 (12.3)	< 0.001

### Association between RAR and short-term mortality

**Figure 1:**
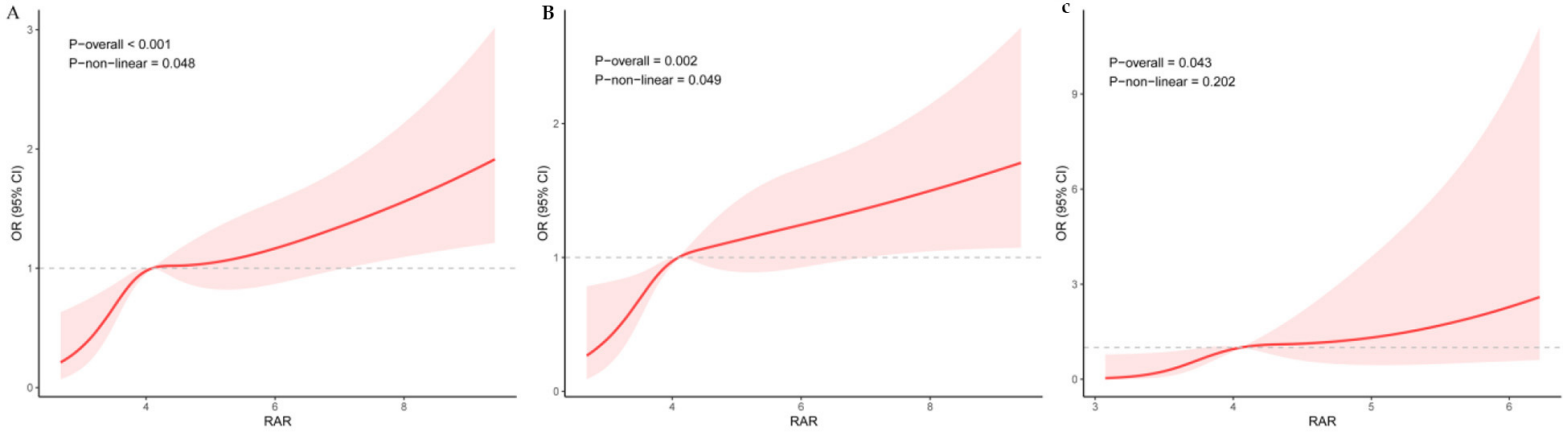
Association between RAR and short-term mortality in critical ICH revealed by restricted cubic spline (RCS) analysis. **(A)**: Relationship of RAR with ICU short-term mortality (internal cohort). **(B)**: Relationship of RAR with in-hospital short-term mortality (internal cohort). **(C)**: Relationship of RAR with ICU short-term mortality (external validation cohort).

Cox regression analysis demonstrated that RAR, as a continuous variable, was significantly and positively associated with the risk of ICU mortality across all three statistical models (Table 2): the unadjusted model (HR = 1.35, 95% CI: 1.28–1.42, *p* < 0.001), the model adjusted for demographics and comorbidities (HR = 1.30, 95% CI: 1.22–1.38, *p* < 0.001), and the fully adjusted model accounting for intergroup differences (HR = 1.17, 95% CI: 1.08–1.27, *p* < 0.001). Similarly, when analyzed as a categorical variable, the high-RAR group consistently showed a significantly elevated risk of ICU mortality compared to the low-RAR group across the same model progression (Model 1: HR = 2.74; Model 2: HR = 2.20; Model 3: HR = 1.53; all *p* < 0.05). Restricted cubic spline (RCS) analysis further revealed a significant non-linear, positive dose-response relationship between RAR and ICU mortality risk (overall *p* < 0.001, non-linear *p* = 0.048), indicating a progressive increase in risk with rising RAR levels (Figure 1A).

**Table 2:** The relationship between RAR and short-term mortality rate in ICU (internal queue).

	Model 1			Model 2			Model 3		
**Characteristic**	**HR**	**95% CI**	***p*** **-value**	**HR**	**95% CI**	***p*** **-value**	**HR**	**95% CI**	***p*** **-value**
**RAR**	1.35	1.28, 1.42	< 0.001	1.30	1.22, 1.38	< 0.001	1.17	1.08, 1.27	< 0.001
RAR group									
Low	Ref	Ref		Ref	Ref		Ref	Ref	
Moderate	1.34	0.96, 1.87	0.087	1.18	0.84, 1.65	0.3	1.13	0.80, 1.58	0.5
High	2.74	2.04, 3.69	< 0.001	2.20	1.62, 2.99	< 0.001	1.53	1.09, 2.15	0.014

CI, Confidence Interval; HR, Hazard Ratio.

A consistent positive association was also observed between RAR as a continuous variable and in-hospital mortality (Table 3), which remained significant after sequential adjustments (Model 1: HR = 1.31; Model 2: HR = 1.29; Model 3: HR = 1.14; all *p* ≤ 0.001). Categorical analysis confirmed that the high-RAR group had a significantly higher risk of in-hospital mortality than the low-RAR group (Model 1: HR = 2.86; Model 2: HR = 2.26; Model 3: HR = 1.52; all *p* < 0.05). The RCS analysis again indicated a significant non-linear, positive dose-response relationship for in-hospital mortality (overall *p* = 0.002, non-linear *p* = 0.049), supporting a progressive increase in risk with higher RAR (Figure 1B).

**Table 3:** The relationship between RAR and short-term mortality rate in hospital (internal queue).

	Model 1			Model 2			Model 3		
**Characteristic**	**HR**	**95% CI**	***p*** **-value**	**HR**	**95% CI**	***p*** **-value**	**HR**	**95% CI**	***p*** **-value**
**RAR**	1.31	1.25, 1.38	< 0.001	1.29	1.22, 1.37	< 0.001	1.14	1.05, 1.23	0.001
RAR group									
Low	Ref	Ref		Ref	Ref		Ref	Ref	
Moderate	1.39	1.00, 1.95	0.053	1.21	0.87, 1.70	0.3	1.05	0.75, 1.48	0.8
High	2.86	2.12, 3.85	< 0.001	2.26	1.66, 3.08	< 0.001	1.52	1.08, 2.15	0.017

CI, Confidence Interval; HR, Hazard Ratio.

### Incremental value of RAR

**Figure 2:**
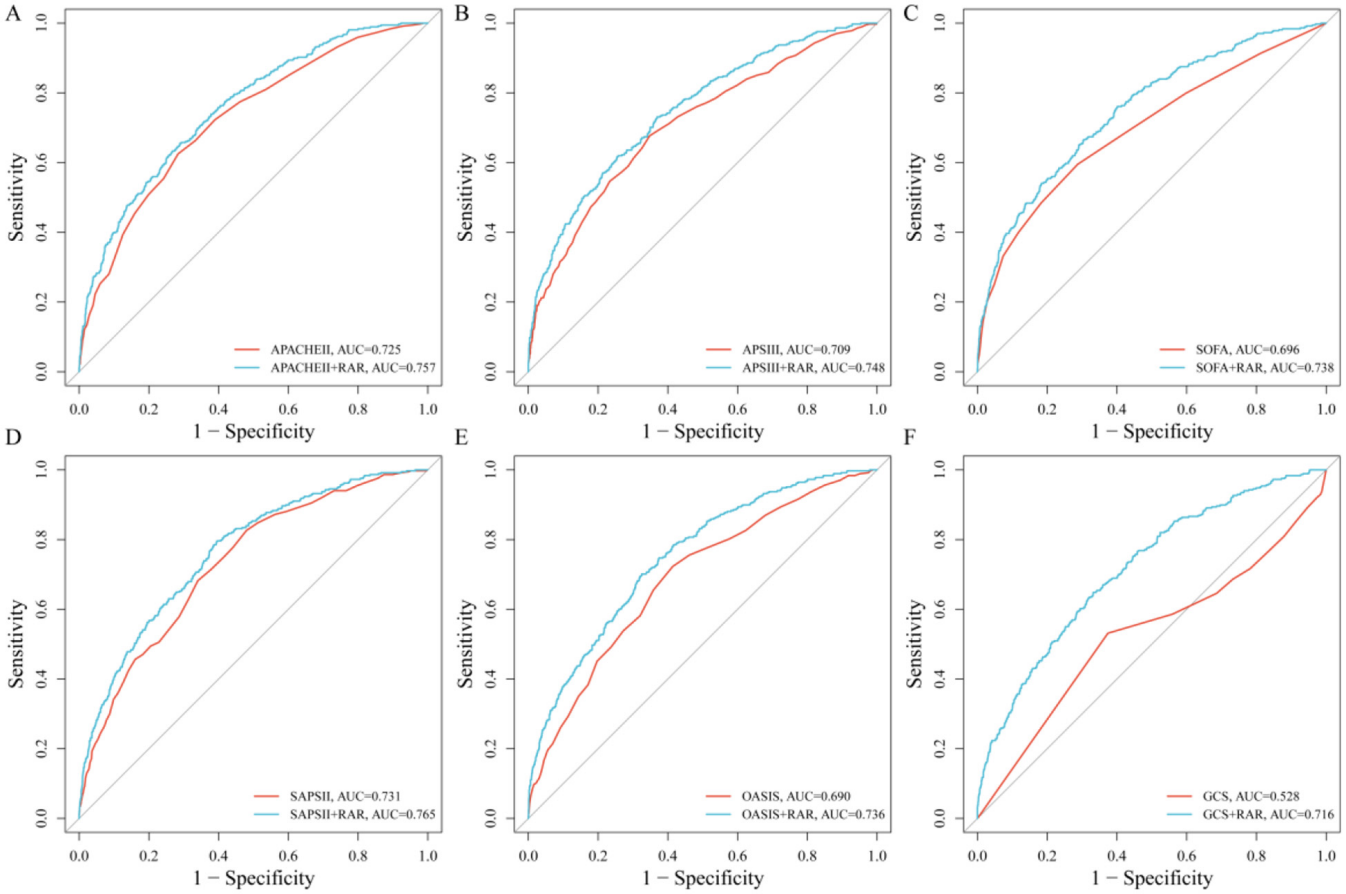
Incremental value of RAR when added to six traditional critical illness scoring systems in the internal cohort. **(A)**: APACHE II. **(B)**: APS III. **(C)**: SOFA. **(D)**: SAPS II. **(E)**: OASIS. **(F)**: GCS.

To evaluate the added predictive value of RAR, we combined it with six established critical illness severity scoring systems (APACHE II, APS III, SOFA, SAPS II, OASIS, and GCS) for outcome prediction. Incorporating RAR consistently improved the predictive accuracy of all scores (Figure 2). Specifically, the area under the curve (AUC) increased from 0.725 to 0.757 for APACHE II, from 0.709 to 0.748 for APS III, from 0.696 to 0.738 for SOFA, from 0.731 to 0.765 for SAPS II, and from 0.690 to 0.736 for OASIS. The most substantial improvement was observed for the GCS score, whose AUC increased markedly from 0.528 to 0.716. Delong's test confirmed that the enhancement in predictive performance from adding RAR was statistically significant for all six scoring systems (all *p* < 0.01). This indicates that RAR provides important incremental prognostic information beyond traditional critical care scores.

### Subgroup and interaction analyses

Subgroup and interaction analyses were conducted to examine the influence of demographic characteristics and comorbidities on the association between RAR and ICU mortality. After multivariate adjustment, a higher RAR remained significantly associated with increased ICU mortality risk in most subgroups (HR > 1, *P* < 0.05; see forest plot). However, this association did not reach statistical significance in patients with COPD (HR = 1.04, 95% CI: 0.84–1.30, *P* = 0.689), CKD (HR = 1.08, 95% CI: 0.86–1.37, *P* = 0.493), or hypertension (HR = 1.12, 95% CI: 0.98–1.27, *P* = 0.088). These subgroups had relatively limited sample sizes and fewer outcome events, which may have reduced statistical power. Therefore, the non-significant findings in these groups should be interpreted with caution, as they may reflect a risk of Type II error rather than a true absence of effect. Formal interaction tests revealed no significant interactions between RAR and any of the tested variables, including age, sex, race, or specific comorbidities (all *P* for interaction > 0.05). This supports a generally consistent predictive effect of RAR across subgroups, with the non-significant results likely attributable to sample size limitations.

### External validation in a real-world cohort

An external validation cohort comprised 472 patients with intracerebral hemorrhage (ICH), with a 28-day mortality rate of 9.96%. In a Cox regression model adjusted for all potential covariates, a higher RAR was significantly associated with an increased risk of 28-day mortality (HR = 1.75; 95% CI: 1.05–2.92, *p* = 0.031). Subsequent RCS analysis confirmed a significant non-linear, positive dose-response relationship between RAR and short-term ICU mortality in this independent cohort (Figure 1C).

### Development and validation of a risk prediction model for ICH patients

**Figure 3:**
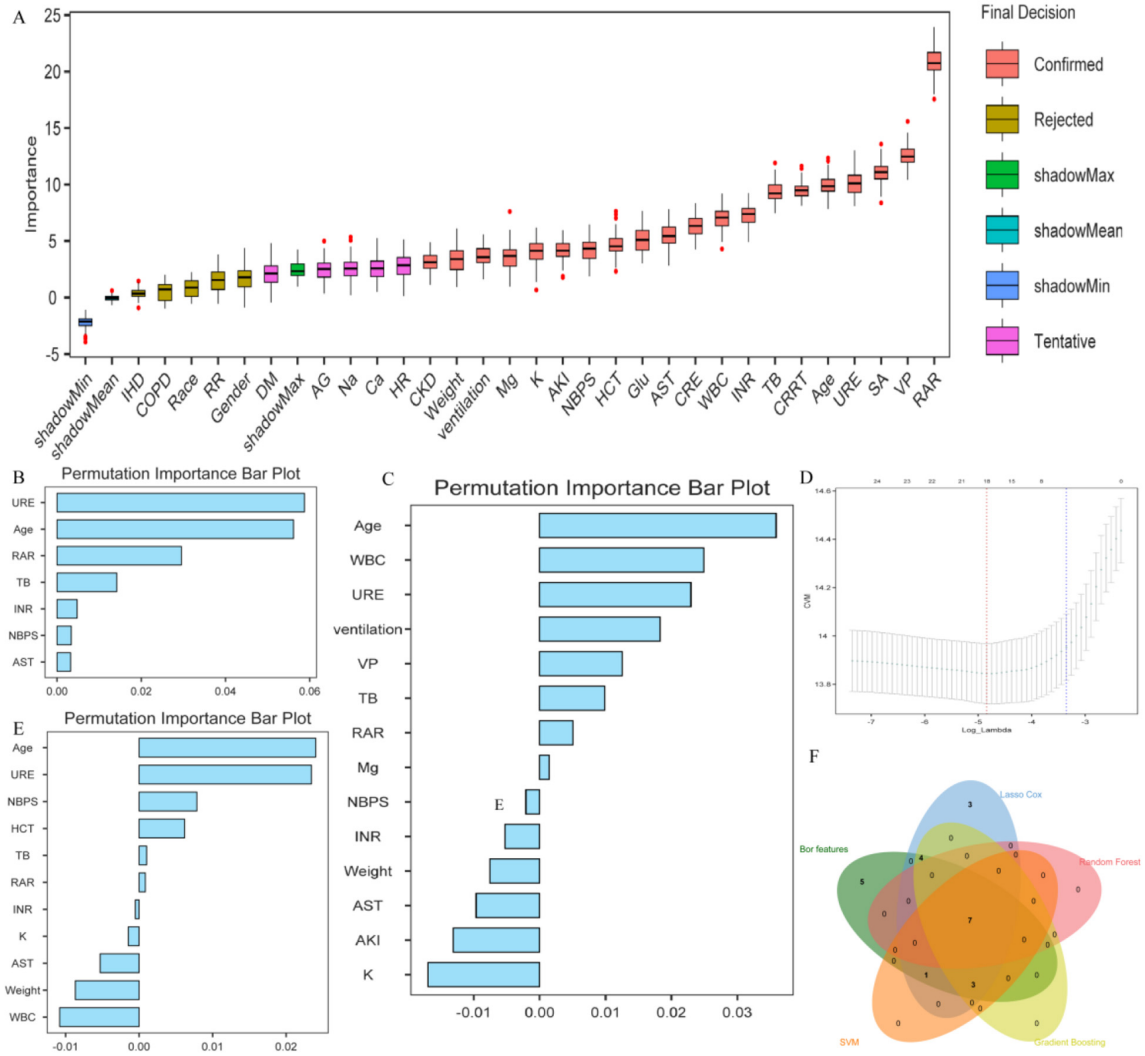
Process for screening core variables closely associated with short-term ICU mortality in critically ill ICH patients. **(A)**: Feature importance ranking by the Boruta algorithm. **(B)**: Features selected by the random forest algorithm. **(C)**: Features selected by gradient boosting. **(D)**: Features selected by LASSO Cox regression. **(E)**: Features selected by the support vector machine algorithm. **(F)**: Venn diagram identifying features common across all five methods.

**Figure 4:**
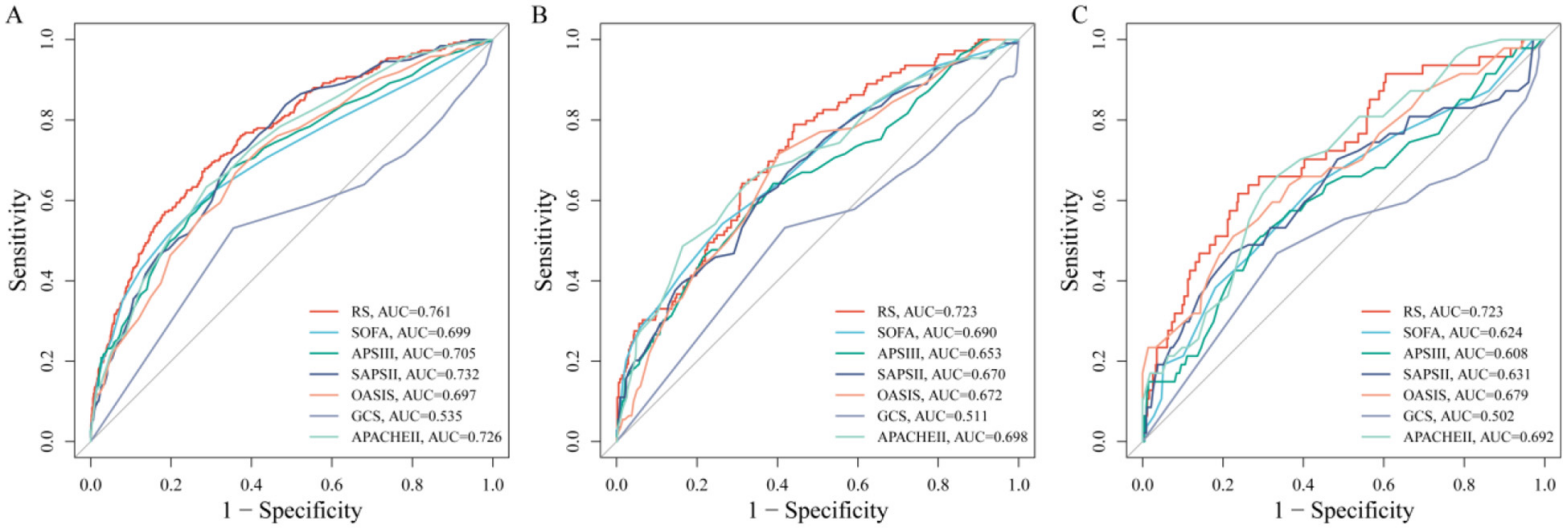
Validation of the simplified risk score model. **(A)**: Internal training cohort. **(B)**: Internal validation cohort. **(C)**: External real-world cohort.

Within the internal training cohort, five machine learning algorithms were used to identify variables closely associated with ICU mortality. The number of identified variables varied by algorithm. A Venn diagram was then used to identify seven key variables shared across all methods: age, RAR, blood urea nitrogen (URE), INR, total bilirubin (TB), AST, and non-invasive blood pressure systolic (NBPS) (Figures 3A–F). These were used to construct a risk assessment model for predicting 28-day mortality in ICH patients. The model formula is: Risk Score = (0.02516424 × Age) + (0.5750546 × RAR) + (0.2552590 × INR) + (0.1139166 × TB) + (0.01042515 × URE) - (0.000003527719 × AST) - (0.005256673 × NBPS). Compared to traditional severity scores (SOFA, APS III, SAPS II, OASIS, GCS, and APACHE II), this model demonstrated higher sensitivity and specificity for predicting 28-day ICU mortality (Figures 4A–C). It achieved an AUC of 0.761 in the internal training cohort (Figure 4A), 0.723 in the internal validation cohort (Figure 4B), and 0.723 in the external real-world cohort (Figure 4C). These consistent results indicate that the model exhibits robust stability and predictive performance across different cohorts.

## Discussion

ICH is a critical condition caused by bleeding from ruptured blood vessels into the brain parenchyma. Globally, there are approximately 3.5 million cases of non-traumatic ICH annually, with high mortality rates reaching up to 40% within the first month and 54% within one year (1–4). Most survivors experience long-term functional and cognitive impairments, placing a substantial burden on families and society. Consequently, early clinical intervention and accurate prognosis assessment are crucial for reducing mortality and improving long-term outcomes. Although several prognostic scoring systems for ICH exist, their clinical utility is often limited by complex metrics and operational impracticality (6–8). In this context, the development of simple, precise biomarkers to facilitate early identification of high-risk patients and guide treatment has become an important direction for improving ICH management.

Within this context, our study demonstrates for the first time that an elevated RAR is an independent risk factor for both 28-day ICU mortality and in-hospital all-cause mortality in ICH patients. This association remained consistent across various demographic characteristics and comorbidities, suggesting RAR is an effective predictive marker for adverse outcomes in this population. Furthermore, we developed and validated a risk prediction model incorporating routine clinical indicators and RAR to estimate 28-day ICU mortality risk. Compared to traditional severity scores, our model demonstrated higher sensitivity and specificity with stable, reliable predictive performance in both internal and external validation cohorts. These findings suggest this model could prompt clinicians to intensify monitoring and more proactively initiate or adjust comprehensive management strategies, including source control of infection, early resuscitation, and organ support.

RDW is a routine hematologic parameter reflecting heterogeneity in red blood cell volume (11, 12). Recent studies have established RDW as a useful marker for assessing systemic inflammation and nutritional status in ICH patients (15, 16, 24, 25). The underlying mechanism involves the systemic inflammatory and oxidative stress responses following ICH. Inflammatory cytokines can disrupt erythropoiesis, interfere with red blood cell maturation, and lead to the release of immature erythrocytes into circulation (11, 12, 26, 27). Furthermore, inflammation may alter red cell morphology and volume distribution by perturbing iron metabolism, reducing erythrocyte membrane stability, and accelerating apoptosis (11, 12). An elevated RDW not only indicates increased red cell destruction but may also exacerbate brain injury by promoting endothelial stress, microcirculatory dysfunction, and secondary cellular apoptosis (24–27).

On the other hand, albumin, the primary plasma protein synthesized by the liver, plays a key role in maintaining osmotic pressure, mitigating oxidative stress, and modulating inflammatory responses (9, 10). Serum albumin level is also a critical indicator of nutritional status and the degree of inflammation (9, 10). In the pathology of ICH, hypoalbuminemia and inflammation engage in a bidirectional vicious cycle: low albumin states are often associated with malnutrition and inflammatory activation, while persistent inflammation further suppresses albumin synthesis, exacerbating nutritional depletion and organ damage (9, 10, 27). Moreover, low albumin levels can impair antioxidant capacity, promote lipid peroxidation, and compromise immune function (25, 28, 29).

Building upon this pathophysiological foundation, an elevated RAR is not a coincidental finding but rather an integrated manifestation of the vicious cycle between systemic inflammation and nutritional/colloid depletion. A high RAR state may directly exacerbate secondary brain injury following ICH and lead to increased short-term mortality through the following mechanisms: First, inflammation-induced elevation of RDW not only reflects impaired erythropoiesis but also signifies reduced erythrocyte deformability. These rigid red blood cells have difficulty navigating through the compressed cerebral microcirculation, resulting in local tissue hypoxia and microcirculatory disturbances, thereby expanding the ischemic penumbra and exacerbating secondary neuronal injury (24, 26, 27). Concurrently, inflammatory cytokines can directly compromise blood-brain barrier integrity, aggravating vasogenic cerebral edema (26, 27). Second, in the setting of hypoalbuminemia, the body's capacity to clear neurotoxic substances, such as iron ions released from cell-free hemoglobin and lipid peroxides, is impaired, leading to accumulation of these toxins in brain tissue and directly promoting apoptosis and lipid peroxidation (28, 29). More importantly, decreased albumin levels diminish the colloid osmotic pressure gradient between the intravascular and extravascular compartments, facilitating fluid extravasation into the brain parenchyma. This exacerbates cerebral edema, leading to elevated intracranial pressure and an increased risk of brain herniation—one of the primary causes of early mortality in ICH patients.

Thus, RAR integrates these two dimensions, precisely capturing the imbalance between “inflammation-driven injury” and “exhaustion of repair capacity”: A high RAR state signifies that, on one hand, a robust inflammatory response (high RDW) is continuously disrupting the blood-brain barrier and inducing microcirculatory dysfunction; on the other hand, the body's endogenous protective mechanisms (low albumin) are overwhelmed and unable to effectively clear toxins or maintain osmotic stability. This dual scenario, simultaneously “fueling the fire” while “diminishing the capacity to extinguish it,” synergistically drives the progression of malignant cerebral edema and the occurrence of systemic complications, ultimately culminating in a sharply increased risk of short-term mortality.

Furthermore, significant differences in baseline characteristics were observed between the two cohorts (Supplementary Table S4). Patients in the external validation cohort were slightly older (67.0 years vs. 65.2 years), had lower RAR levels (4.21 vs. 4.34), higher hemoglobin concentrations, lower white blood cell counts, and a markedly lower 28-day ICU mortality rate compared to the derivation cohort (10.0% vs. 15.8%). These differences may be attributable to variations in population characteristics between China and the United States, disparities in healthcare systems, and advances in care standards over the intervening decade. Despite this heterogeneity, the predictive model demonstrated stable discriminative ability in the external validation (AUC = 0.723), which was entirely consistent with the internal validation results. This finding robustly supports the model's strong cross-setting generalizability—its core variables effectively capture the mortality risk driven by “inflammation-nutrition imbalance” across diverse populations, healthcare systems, and temporal contexts. Notably, RAR maintained significant predictive value even within the low-risk population (HR = 1.75), further supporting its potential as a universal prognostic marker. Future studies should validate the model's calibration in multicenter prospective cohorts.

It is noteworthy that, compared to prior studies focusing primarily on long-term survival (22), our study design and validation offer distinct advantages. First, by focusing on ICU and hospital outcomes within 28 days, we target the critical, guideline-recommended window of intensive intervention that determines short-term survival and functional status. This shorter observation period minimizes interference from post-discharge confounders, allowing the association between RAR and prognosis to more clearly reflect the direct impact of acute-phase pathophysiology. Second, to enhance generalizability and robustness, we independently validated our initial findings using an external real-world patient cohort from a different care period. This multi-center, cross-regional validation strategy strongly supports the reproducibility and broad applicability of the association between RAR and short-term outcomes in critical ICH, reducing the risk of chance findings or selection bias inherent to single-center studies. Finally, building on our findings, we integrated readily available clinical indicators to construct and validate a concise, practical risk prediction model. Demonstrating good discriminative ability and stability in both internal training and external validation sets, this model provides a actionable tool for early risk stratification, intensified monitoring, and personalized management of critically ill ICH patients, facilitating the direct translation of research findings into clinical practice.

Furthermore, compared with traditional scoring systems such as APACHE II, SOFA, and SAPS II, RAR offers the following key advantages: First, it requires only two routine laboratory parameters, circumventing the complexity and data acquisition challenges associated with conventional scoring systems. Second, it directly targets the core pathological axis of secondary brain injury following ICH, namely, inflammation and nutritional depletion—thereby addressing the limitation of traditional scores, which primarily reflect systemic organ dysfunction and lack sensitivity to intracranial-specific events. Third, RAR demonstrated robust predictive performance in both internal and external cohorts; it not only remained independent of traditional scoring systems but also significantly enhanced their predictive capacity (AUC improvement: 0.016–0.188), providing a unique prognostic dimension—inflammation–nutrition imbalance, that is not captured by existing tools.

While this study provides clinically meaningful findings and a potentially useful risk prediction model, several limitations must be acknowledged. First, all data for the independent real-world cohort came from a single tertiary hospital with a limited sample size, which may affect the generalizability of the results. Second, despite rigorous adjustment for known confounders, the lack of neuroimaging data (e.g., hematoma location/volume, cerebral edema) is a significant limitation. Unmeasured initial neurological injury severity is a potent confounder that may substantially influence the observed associations, thereby tempering confidence in the conclusion of RAR's independence. Third, evolving definitions of sepsis based on updated clinical guidelines may introduce variability in case inclusion and comparability across different time periods. Furthermore, RAR was calculated from a single static measurement at admission, precluding assessment of its dynamic changes during treatment and their prognostic value. Finally, given the retrospective observational design, our study cannot establish a causal relationship between RAR and adverse outcomes in ICH patients. Based on these limitations, future large-scale, multi-center, prospective studies with more comprehensive clinical data are warranted to further validate the prognostic value and clinical utility of RAR in this population.

In summary, RAR is a composite parameter derived from two routine laboratory indices and holds unique clinical value in practice. First, RAR can be utilized for emergency triage and early warning, with results obtainable within 24 h of admission. For patients with a significantly elevated RAR, clinicians should remain vigilant regarding the potential risk of deterioration, even if traditional scores are within an acceptable range, and consider early transfer to the ICU or enhanced monitoring. Second, RAR offers significant cost-effectiveness advantages: both RDW and albumin are routinely measured upon ICU admission, and calculating RAR incurs no additional medical expense. It can be automatically generated within electronic medical record systems, saving valuable clinical time. Furthermore, RAR may serve as a stratification tool for clinical study enrollment, helping to identify high-risk populations for anti-inflammatory or nutritional intervention trials. As a zero-cost, readily available, and dynamically monitorable biomarker, RAR demonstrates strong potential for implementation across diverse healthcare settings with varying resource availability.

## Conclusion

This study confirms that the RAR is an independent predictor of 28-day ICU mortality and in-hospital all-cause mortality in intracerebral hemorrhage patients, exhibiting a non-linear, positive association with mortality risk. By integrating RAR with routine clinical indicators, we developed and validated a clinically practical risk prediction model with good discriminative performance (AUC 0.723–0.761 in internal and external validation). This model outperformed traditional critical illness scores in stably identifying high-risk patients, facilitating early targeted intervention. Despite limitations including its retrospective design, single-center external validation, and lack of neuroimaging parameters, this study provides a novel biomarker and a practical tool for ICH prognosis assessment. Future multi-center prospective studies are needed to further validate its clinical value and applicability.

## Data Availability

The original contributions presented in the study are included in the article/supplementary material, further inquiries can be directed to the corresponding authors.
